# Does Breast Feeding Protect Mothers From Obesity?

**DOI:** 10.7759/cureus.70217

**Published:** 2024-09-25

**Authors:** Süleyman Coşgun, Derya Ünal, Aysun Çalışkan Kartal, Osman Bedir

**Affiliations:** 1 Internal Medicine, Gastroenterology, Kütahya University of Health Sciences, Kütahya, TUR; 2 Internal Medicine, Kütahya University of Health Sciences, Kütahya, TUR; 3 Gastroenterology and Hepatology, Kütahya University of Health Sciences, Kütahya, TUR; 4 Gastroenterology, Kütahya University of Health Sciences, Evliya Celebi Education and Research Hospital, Kütahya, TUR

**Keywords:** body mass index, breastfeeding, fibrosis score, hepatosteatosis, steatosis nash

## Abstract

Background: Hepatosteatosis, which we frequently observe today with change in lifestyle, is often unnoticed, but preventable and reversible; if not prevented, it can lead to serious comorbidities. There is contradicting evidence in the literature; we believe that breastfeeding has a protective effect on hepatosteatosis. In this cross-sectional study we conducted, we aimed to examine the relationship between breastfeeding duration, metabolic parameters and fatty liver.

Methods: We examined the data of 135 patients aged 20-40 years who have had at least one pregnancy and were admitted to our polyclinic. Forty-five healthcare staff who never breastfed were included in the control group. Measurements of height and weight were taken, and number of children and total breastfeeding time were questioned. Blood values were measured to calculate insulin resistance, non-alcoholic fatty liver disease (NAFLD) fibrosis score and Fibrosis-4 (FIB-4) score.

Results: Consequently, there was no significant correlation between total breastfeeding time and body mass index (BMI), NAFLD fibrosis score, Homeostatic Model Assessment for Insulin Resistance (HOMA-IR) value and hemoglobin A1C (HbA1C). When two groups were formed as patients breastfeeding for less than six months and patients breastfeeding for more than six months, a significant difference in BMI was observed between these two groups (p: 0.02). There was a significant relationship between BMI and NAFLD (p: 0.00) and HOMA-IR (p: 0.00). It was observed that there was a significant difference between BMI FIB-4 and NAFLD fibrosis scores of the control group and breastfed group.

Conclusions: Lactation should be maintained for at least six months for maternal health together with the baby’s health, and more comprehensive studies should be conducted for long-term data.

## Introduction

Increasing worldwide obesity and associated diabetes and hepatosteatosis is a major health problem and occupies an important space in the daily practice of hepatology. The effective treatment of viral hepatitis with antiviral treatments and the acceleration of vaccination strategies with the progression of preventive medicine possibly cause a shift from viral hepatitis, which is prominent in the etiology of cirrhosis, to non-alcoholic steatohepatitis (NASH). In addition to the increase in visceral adipose tissue, the content of free fatty acid in the portal area increases in the liver. Consequently, the ratio of fatty acid released from the liver decreases [1]. Hepatosteatosis results in different clinical images based on the percentage of affected hepatocytes [2]. Non-alcoholic fatty liver disease (NAFLD) occurs when > 5% of hepatocytes are affected [2,3]. Its prevalence varies between different societies. In the later period, inflammation and lipotoxicity forms the underlying basis of transformation of simple steatosis in the liver into steatohepatitis [2,4]. This clinical situation is called NASH. In advanced stages, 20% of patients can develop progressive fibrosis and cirrhosis and hepatocellular cancer (HCC) can be seen [2-7]. Comorbid conditions, particularly the presence of diabetes mellitus (DM), accelerate this process [8]. The prevalence of NAFLD and NASH is between 3% and 50% in different studies [6]. NAFLD, which is associated with metabolic syndrome, can therefore develop on the basis of insulin resistance, particularly in the background of type 2 DM [6, 8]. The most common age group is 50-60 years old. Although not reported in every study, being a woman is a risk factor, and this condition is more common in the Hispanic population [6]. Certain studies note that the male gender is a risk factor [4,9]. It is associated with obesity and hypertriglyceridemia caused by a sedentary life [10]. Previous studies have emphasized that conditions such as polycystic ovary syndrome, dyslipidemia and obstructive sleep apnea syndrome are associated with NAFLD [4]. According to World Health Organization (WHO) data in 2016, >650 million obese individuals were detected worldwide [11]. 2017 data published in Turkey indicate that the prevalence of being overweight was 66.9%. With respect to sex, this prevalence is 70% in women and 63.7% in men [12]. The prevalence of NAFLD in Turkey is estimated to be ~30% [13]. Hepatitis C and Human Immunodeficiency Virus (HIV) can be included as risk factors that cause the disturbance of metabolic balance. There are suspicions that it may increase the risk of primary aldosteronism and myotonic dystrophy [5]. An inverse correlation has been reported between the severity of NAFLD and vitamin D level [9]. There are patients monitored for NAFLD who are not obese (BMI < 23 kg/m2) [9]. Here the effect of waist circumference and triglyceride levels comes to the fore. Although obesity is a risk factor for NAFLD, a relationship has been shown between sarcopenia and hepatic fibrosis in the progressive process leading to cirrhosis [7,14]. Insulin resistance forms the basis of NAFLD but genetic effects are also monitored [2]. The gene whose effect is considered to be the highest is PNPLA3 [15]. Melanocortin-3, hepatic triacyl glycerol (HTAG), and TM6SF2 mutations are present [4,10]. Insulin resistance most often occurs as part of metabolic syndrome and is diagnosed in the period of diabetes and prediabetes [2]. Excess calories disrupt the signaling pathway, which begins with the insulin receptor [8]. A study showed that insulin-like growth factor-1 (IGF-1) levels in the NAFLD group were low and IGF-1 and insulin levels were the predictors of NAFLD [16]. Furthermore, IGF-1 was lower in the group with impaired liver function [16]. This clinical image develops in the normal course of pregnancy, and its course varies as per many factors that continue to be investigated in the postpartum period [17,18]. Based on this information, our aim was to investigate whether insulin resistance developed in the patients after pregnancy and examine the effect of breastfeeding on insulin resistance and NAFLD during this period.

This article was previously posted to the medRxiv preprint server on November 23, 2022.

## Materials and methods

Female patients who were diagnosed with fatty liver by their family physician or internal medicine outpatient clinic and referred to our outpatient clinic were included in the study. We examined the data of 135 patients aged 20-40 who had at least one pregnancy. Informed consent was obtained from each patient participating in the study, and routine examinations were performed on female patients admitted due to fatty liver. The degree of fatty liver on upper abdominal ultrasonography (grade I-III) and the number of healthy children born and the duration of breastfeeding periods were noted. Height and weight measurements were taken and BMI was calculated. The patients were asked when they had their last child, and the time from that birth to the evaluation date was recorded. Forty-five healthcare staff who never breastfed were included in the control group. All variables were analyzed for normality. Parametric tests were used for normally distributed variables and nonparametric tests were used for nonnormally distributed variables. T and Mann-Whitney U tests were used for comparing independent variables between the two groups. For three or more groups, ANOVA test and Kruskal-Wallis test were used, and Pearson’s and Spearman’s correlation tests were used to determine the relationship between two variables. Both univariate and multivariate logistic regression analyses were used to determine risk factors for osteopenia and osteoporosis. p < 0.05 was considered to be statistically significant. The NAFLD score and Fibrosis-4 (FIB-4) score, which were used in the evaluation, were calculated with the obtained data. All the procedures in studies involving human participants were performed in accordance with the ethical standards of the institutional and/or national research committee and with the 1964 Helsinki Declaration and its later amendments or comparable ethical standards.

## Results

The mean age of 135 female patients included in the study was 34.4 ± 4.8 (21-40) years, the mean number of children was 1.8 ± 0.6 (1-3), and the mean total breastfeeding time was 25 ± 16 (0-72) months. The time from the last birth to the evaluation date was 7.4 ± 5.4 (0-22) years. Given the first six-month period, which is an important threshold for baby health in breastfeeding, patients were divided into two groups as those breastfeeding for less than six months and those for more than six months. BMI, NAFLD and HOMA-IR were evaluated with respect to breastfeeding periods. Consequently, there was no significant correlation between total breastfeeding time and BMI, NAFLD fibrosis score, HOMA-IR value and HbA1c. When two groups were formed as patients breastfeeding for less than six months and patients breastfeeding for more than six months, a significant difference in BMI was observed between these two groups (p: 0.02). BMI was 27.2 ± 5.3 in mothers breastfeeding for less than six months while it was 30.6 ± 6.6 in mothers breastfeeding for more than six months. There was a significant relationship between BMI and NAFLD (p: 0.00) and HOMA-IR (p: 0.00). Therefore, the development of insulin resistance of women giving birth helps the mother in the first six months with breastfeeding and the result suggests that it has a positive effect on weight loss and liver steatosis. When the two groups were formed based on breastfeeding for 12 months, no significant difference was reported. According to these results, it seems that the first six months are important for the mother as much as the baby. It was observed that there was a significant difference between BMI (22.7 versus 27.6; p:0.00), FIB-4 (0.51 versus 0.65; p:0.00) and NAFLD fibrosis scores (-3.3 versus -2.4; p:0.00) of the control group and the breastfed group (Table 1).

**Table 1:** BMI difference between mothers who breastfed for less than six months and mothers who breastfed for more than six months, and comparison of BMI, FIB4 and NAFLD fibrosis score between all breastfeeding mothers and the control group. *p:0.002 **p:0.000 ***p:0.002 and ****p:0.000, BMI: body mass index, Fibrosis-4 (FIB-4): index for liver fibrosis is a non-invasive scoring system based on several laboratory tests, non-alcoholic fatty liver disease (NAFLD) fibrosis score: a noninvasive system that identifies liver fibrosis in patients with hepatosteatosis

Groups	BMI	FIB-4	NAFLD fibrosis score
Mothers breastfeeding for <6 months	30.6*	0.68	-1.9
Mothers breastfeeding for >6 months	27.2*	0.65	-2.4
All breastfeeding mothers	27.6**	0.65***	-2.4****
Control group	22.7**	0.51***	-3.3****

With these results, we demonstrated that there is a correlation between BMI and NAFLD and HOMA-IR. Moreover, we observed a correlation between the number of children and FIB-4 score (p: 0.05).

## Discussion

Dietary items received above the daily calorie requirement are stored in the adipose tissue and liver. Increased steatosis in the liver and the subsequent hepatocellular ballooning may lead to NASH, which is accompanied by inflammation, and can progress into fibrosis and cirrhosis. NAFLD occurs as a result of >5% of hepatocytes being affected [2,3]. In 20% of cases, NASH developing with concomitant inflammation can progress into cirrhosis and HCC in advanced stages [2,4-7]. Outside HCC, there is an increased risk of extrahepatic cancer [19-21]. NAFLD is examined in four groups. Grade 0 refers to the normal liver. If the number of monitored hepatocytes with steatosis is <5%, the classification is Grade 1. If it is between 5% and 33%, the classification is Grade 2; if it is between 33% and 66%, the classification is grade two-three. NASH is the condition when lobular inflammation and ballooning occur in addition to hepatosteatosis. Mortality increases in F2 and F3 before cirrhosis develops [22]. Disease activity is estimated with NAFLD activity score (NAS): 0-3 points for steatosis, 0-3 points for lobular inflammation, and 0-2 points for ballooning. Similarly, steatosis (S), activity (A), and fibrosis (F) are investigated with the SAF score [3]. The risk of developing DM in the later period in patients with gestational DM is seven times or more than in a non-gestational DM patient [23]. In the prospective study of Gunderson et al. covering a two-year period, the relationship between lactation and DM was reported [24]. In many studies, a clear relationship between breastfeeding and type 2 DM and postpartum weight loss was observed [25,26]. There is evidence that the activation of lactation and increase in duration reduces metabolic parameters and the formation of associated diseases [27,28]. In this study, we observed that the breastfeeding period effectively reduces insulin resistance, particularly in mothers who have completed six months. This can be interpreted as breastfeeding reducing the risk of DM in nursing mothers. In a study where measurements in pregnant women were made from the beginning of pregnancy until the postpartum period, the effect of lactation on weight loss during the postpartum period was noted [29]. The overweight group included mothers breastfeeding for shorter time periods [28]. In another study, ensuring complete breastfeeding is effective for weight loss in the postpartum period [30]. The effect of the number of births on weight gain should be considered. Although the weight gain is usually around the abdomen, the weight lost in the initial postpartum period may not be abdominal fat because of the effect and duration of breastfeeding [28,29]. One study noted that weight loss was from the abdomen and thighs [31]. Consistent with our results, higher insulin, insulin resistance, glucagon, cortisol, triacylglycerol, epinephrine and dopamine levels were monitored in nursing mothers compared to non-nursing mothers [32,33]. Factors affecting postpartum weight are not only breastfeeding and the diet applied after childbirth but also include prenatal weight, lifestyle and the target during childbirth [34]. There are studies that suggest that the main factor is psychological [35]. Particularly important is the first half of pregnancy [34]. In the first 30 days, there is significant weight loss in women who are obese before pregnancy; however, as this period extends to 360 days, weight loss is significant in people who are underweight before pregnancy [26]. Most studies report that weight loss begins with at least three months of breastfeeding; this difference becomes more evident with six months of breastfeeding [26,30,31,36]. From the reverse perspective, those who gained more weight than they intended during pregnancy and those who were obese before pregnancy were more likely to stop postpartum breastfeeding [35,37]. Moreover, mothers who exercise more and smoke less often breastfeed longer [25]. In the black race, the waist circumference was observed to be wider in primiparous women, pregnant women with high prenatal weight, and mothers breastfeeding for shorter time periods. However, the difference in waist circumference and BMI with respect to breastfeeding was not statistically significant in this study [38]. Difference in prolactin response in obesity may have an effect on breastfeeding [38]. High greline and peptide YY were reported to be associated with patients who were monitored during lactation [39]. The hormone that promotes the proliferation of β cells in pregnancy is prolactin [24]. Prolactin varies in proportion to adipose tissue and is known to act on carbohydrate metabolism with fat [40]. Pioglitazone can be used in treatment [9,15]. Vitamin E is an effective antioxidant; however, because of its side effects, it has to be used with caution and a study indicating the dose range should be conducted [21]. The most effective treatment is diet and weight loss, weight loss ensures both NAFLD and NASH regression, as well as stopping progression in cases with fibrosis development [15]. Protein-weighted nutrition in the diet was reported to be more effective in the regression of hepatosteatosis [8]. Metformin has a weak effect of reversing hepatosteatosis and preventing the formation of HCC [15]. Ursadeoxycholic acid caused an increase in low-density lipoprotein (LDL) levels [15]. While there are different studies on sodium-glucose co-transporter 2 (SGLT2) inhibitors, there are studies where aspartate aminotransferase (AST), gamma-glutamyl transferase (GGT) and steatosis declined [8]. Studies on novel molecules are ongoing [8]. In conclusion, lactation should be maintained for at least six months for maternal health together with the baby’s health, and more comprehensive studies should be conducted for long-term data. The limitations of our study were that the duration of breastfeeding was learned from anamnesis, the number of patients participating in the study was limited, and fatness was evaluated by ultrasonography.

## Conclusions

We could not detect a direct correlation of total breastfeeding duration in the postpartum period with body mass index and hepatosteatosis. However, breastfeeding for longer than six months was associated with a significant reduction in BMI compared to shorter duration. A decrease in the meaning of BMI was also found to be associated with hepatosteatosis.
